# Systematic review of the management of perioperative tube-fed enteral nutrition in esophageal cancer patients

**DOI:** 10.3389/fnut.2026.1717313

**Published:** 2026-07-24

**Authors:** Xinli Wan, Huarong Zheng, Jiayi Yang, Yan Zang, Hailing Li, Lizhen Qin, Caixia Liu

**Affiliations:** 1 Department of Cardiothoracic Surgery, Yichang Central People’s Hospital, Yichang, Hubei, China; 2 Department of Thoracic Surgery, The First Hospital of China Medical University, Shenyang, Liaoning, China; 3 Department of Gastrointestinal Surgery, Yichang Central People’s Hospital, Yichang, Hubei, China

**Keywords:** enteral nutrition, esophageal cancer, perioperative care, tube feeding, nutrition management, evidence-based nursing

## Abstract

**Aim:**

To summarize the available evidence on perioperative tube-fed enteral nutrition in esophageal cancer patients.

**Design:**

This article was an evidence summary according to the evidence summary reporting standard of the Fudan University Center for Evidence-Based Nursing.

**Method:**

A systematic literature search was conducted on the management of tube-fed enteral nutrition in patients with esophageal cancer. The types of literature included clinical decisions, guidelines, expert consensus, evidence summaries, and systematic evaluations.

**Data sources:**

The following databases and resources were systematically searched: BMJ Best Practice, Up To Date, Guidelines International Network, National Guideline Clearing-house, National Institute for Health and Care Excellence, Scottish Intercollegiate Guidelines Network, Registered Nurses’ Association of Ontario, Yi Mai Tong Guidelines Network, The Cochrane Library, PubMed, Web of Science, CINAHL, Embase, Wan Fang, CNKI, VIP, and SinoMed; and websites of relevant professional associations, including National Comprehensive Cancer Network, American Cancer Society, European Society for Clinical Nutrition and Metabolism website, American Society for Parenteral and Enteral Nutrition website, China Nutritional Sciences Resource Platform, and Chinese Nursing Association. The search period spanned from the database setup to November 2024.

**Results:**

A total of 20 articles were included, including 2 clinical decisions, 9 guidelines, 7 expert consensus, 1 evidence summary, and 1 systematic review. A total of 40 pieces of evidence were summarized from 9 aspects: team management, nutritional risk screening and assessment, nutritional support indications, enteral nutritional support routes and timing, target nutrient requirements, enteral nutritional preparations, implementation of tube-fed enteral nutrition, maintenance of tubing, monitoring of nutritional support, and evaluation of outcomes.

**Conclusion:**

This article summarizes evidence on perioperative tube-fed enteral nutrition management in patients with esophageal cancer. Clinical practitioners should select and apply evidence, considering actual medical conditions and the patient’s specific condition, to scientifically administer perioperative tube-fed enteral nutritional support in patients with esophageal cancer.

**Systematic review registration:**

identifier ES20246670.

## 1 Introduction

From 2010 to 2019, esophageal cancer ranked as the seventh most common type of cancer globally and the sixth leading cause of cancer-related deaths worldwide (1). In China, esophageal cancer incidence rates are among the top five globally (2). For localized esophageal cancer, surgical resection remains the primary curative treatment (3). Additionally, postoperative enteral nutrition (EN) improves patients’ nutritional status and reduces the incidence of pulmonary infections and anastomotic leaks (4). However, it also shortens postoperative hospital stays (5). Furthermore, preoperative EN support enhances postoperative nutritional parameters and accelerates postoperative recovery (6).

Although Enhanced Recovery After Surgery (ERAS) protocols advocate early resumption of oral intake, concerns over delayed anastomotic leaks lead many medical centers to postpone oral feeding until 2–4 weeks postoperatively (7). While postoperative tube feeding typically lasts ≤90 days, approximately 23.5% of patients require EN support beyond this period (8). As such, jejunostomy tubes or nasoenteric tubes are commonly used routes for postoperative EN delivery in esophageal cancer patients (9).

Tube feeding plays an indispensable role in perioperative management due to treatment-induced secondary dysphagia. By bypassing the esophageal region, tube feeding ensures direct delivery of nutrients to sites of digestion and absorption (10). Although guidelines, expert consensuses, and organizational standards related to EN exist internationally, along with nursing professionals prioritizing EN management in esophageal cancer, current research predominantly focuses on early postoperative oral feeding, management of EN intolerance, home-based EN support, and general perioperative nutritional management (11–14).

Therefore, this article employed evidence-based methodology to address the following question: What is the available evidence on perioperative tube-fed EN for patients with esophageal cancer? To assess this, we extracted and synthesized relevant evidence to inform the development of a scientifically rigorous protocol for perioperative EN management in this population.

## 2 Materials and methods

### 2.1 Study design

According to JBI, evidence summaries were registered through the JBI Collaborating Center for Evidence-Based Nursing at Fudan University (registration number: ES20246670). This evidence summary followed the methodology for evidence summary development established by the Fudan University Center for Evidence-Based Nursing (15).

### 2.2 Problem establishment

Evidence-based, structured questions were identified using the PIPOST model (16): What is the available evidence on perioperative tube-fed EN for patients with esophageal cancer? The target population for applying evidence (Population, P) was patients undergoing esophageal cancer surgery. The intervention (I) comprised tube-fed EN measures, including nutritional assessment, screening, support, and monitoring. The professionals applying evidence (Professional, P) included the healthcare professionals. The primary outcome indicators (O) were nutritional support compliance rate, complication rate, morbidity and mortality rate, etc.; secondary outcome indicators were hospital days, readmission rate, and knowledge and implementation rates of perioperative tube-fed EN support protocols for esophageal cancer patients among healthcare professionals, etc. The setting for implementing evidence-based practices (setting, S) was the thoracic surgery department or other wards where patients with esophageal cancer are admitted. Type of evidence (T) encompassed clinical decisions, guidelines, expert consensus, evidence summaries, and systematic reviews.

### 2.3 Evidence retrieval

A systematic top-down retrieval of sources of evidence was based on the ‘6S’ evidence resource pyramid model (17). The databases searched included BMJ Best Practice, Up to Date, Guidelines International Network (GIN), National Guideline Clearing-house (NGC), National Institute for Health and Care Excellence (NICE), Scottish Intercollegiate Guidelines Network (SIGN), Registered Nurses’ Association of Ontario (RNAO), Yi Mai Tong Guidelines Network, The Cochrane Library, PubMed, Web of Science, CINAHL, Embase, Wan Fang Database, CNKI, VIP Database, and SinoMed. Searched professional association websites included the National Comprehensive Cancer Network (NCCN), the American Cancer Society (ACS), the European Society for Clinical Nutrition and Metabolism (ESPEN), the American Society for Parenteral and Enteral Nutrition (ASPEN), the China Nutritional Sciences Resource Platform, and the Chinese Nursing Association. Moreover, references to some of the relevant literature were retrieved manually.

The search terms in English and Chinese combined MeSH terms and free text, with Boolean logic (AND/OR) applied to three concept blocks: “esophageal neoplasms/esophagus neoplasm/cancer of esophagus/esophagus cancer/esophageal cancers/esophagectomy/esophagectomies/esophageal surgery/esophageal resection” “EN/enteral feeding/nutrition therapy/nutritional support/artificial feeding” “guideline/consensus/meta-analysis/system review/evidence summary/best practice.” The search period ran from the establishment of the databases to 30 November 2024.

Prior to formal screening, several key target articles were identified, and the search strategy was confirmed to retrieve these articles, serving as a sensitivity check. The full search strategy for PubMed is presented in Table 1, and complete search strategies for all other databases are provided in Supplementary File.

**Table 1:** PubMed search strategy.

Database	No	Search strategy
PubMed	#1	(((((((((((((((esophageal neoplasms[MeSH Major Topic]) OR (esophageal neoplasms[Title/Abstract])) OR (Esophageal Neoplasm[Title/Abstract])) OR (Esophagus Neoplasm[Title/Abstract])) OR (Esophagus Neoplasms[Title/Abstract])) OR (Cancer of Esophagus[Title/Abstract])) OR (Esophageal Cancer[Title/Abstract])) OR (Esophageal Cancers[Title/Abstract])) OR (Cancer of the Esophagus[Title/Abstract])) OR (Esophagus Cancer[Title/Abstract])) OR (Esophagus Cancers[Title/Abstract])) OR (esophagectomy[MeSH Major Topic])) OR (esophagectomy[Title/Abstract])) OR (Esophagectomies[Title/Abstract])) OR (esophageal surgery[Title/Abstract])) OR (esophageal resection[Title/Abstract])
	#2	(((((((((((((enteral nutrition[MeSH Major Topic]) OR (enteral nutrition[Title/Abstract])) OR (Tube Feeding[Title/Abstract])) OR (Enteral Feeding[Title/Abstract])) OR (Gastric Feeding Tubes[Title/Abstract])) OR (Gastric Feeding Tube[Title/Abstract])) OR (Force Feeding[Title/Abstract])) OR (Force Feedings[Title/Abstract])) OR (nutrition therapy[MeSH Major Topic])) OR (nutrition therapy[Title/Abstract])) OR (Medical Nutrition Therapy[Title/Abstract])) OR (Nutritional Support[MeSH Major Topic])) OR (Nutritional Support[Title/Abstract])) OR (Artificial Feeding[Title/Abstract])
	#3	(((((guideline[Title/Abstract]) OR (consensus[Title/Abstract])) OR (meta-analysis[Title/Abstract])) OR (system review[Title/Abstract])) OR (evidence summary[Title/Abstract])) OR (best practice[Title/Abstract])
	#4	#1 AND #2 AND #3

### 2.4 Inclusion and exclusion criteria

*Inclusion criteria*: (1) target population of esophageal cancer patients aged ≥18 years old; (2) the evidence summary was about nutritional screening, assessment, intervention, and evaluation of postoperative nutrition in esophageal cancer patients; (3) the type of literature included was clinical decision, guidelines, expert consensus, evidence summaries, and systematic review; (4) the language of the literature was limited to Chinese or English; if the relevant literature had been updated, the latest version was included.

*Exclusion criteria*: (1) articles were conducted on adolescents or children only; (2) the type of article was guideline interpretation, conference report, or direct translation of foreign guideline versions; (3) duplicated publications or literature for which the full text was unavailable; and (4) literature of lower quality.

### 2.5 Literature screening and quality evaluation

#### 2.5.1 Literature screening process

A systematic literature search was conducted by one researcher across the databases and guideline websites listed in Section 2.2. All retrieved records were imported into reference management software for deduplication.

Two researchers independently screened the titles and abstracts of all retrieved records against the pre-defined inclusion criteria. Irrelevant records were excluded. The full texts of all potentially eligible articles were then retrieved and independently assessed by the same two researchers.

At both the title/abstract screening and full-text review stages, any disagreements between the two reviewers were resolved through discussion. If consensus could not be reached, a third researcher was consulted to make the final decision.

The detailed results of the literature selection process, including the number of records identified, excluded, and included at each stage, are presented in the Results section.

#### 2.5.2 Quality evaluation process

The methodological quality of the included literature was assessed independently by reviewers who had completed systematic evidence-based training. Different assessment tools were utilized based on the type of literature. Four reviewers independently evaluated each guideline using the AGREE II instrument (18). Two reviewers independently assessed each systematic review using the 2016 version of the JBI Center for Evidence-Based Healthcare’s authenticity assessment for systematic review articles (19). Two reviewers independently evaluated each expert consensus article using the 2016 version of the JBI Center for Evidence-Based Healthcare’s authenticity assessment for opinion and consensus articles (20). Clinical decisions from *BMJ Best Practice* and *UpToDate* were considered high-quality evidence and were not subject to further quality evaluation. To obtain evidence from sources such as clinical decisions, recommended practices, and evidence summaries, we traced the primary studies and evaluated them using the appropriate JBI tools with two reviewers. For all assessments, disagreements between the two primary reviewers were resolved through discussion or by consulting a third reviewer.

### 2.6 Evidence classification and recommendation level judgment

Two researchers conducted evidence extraction and synthesis. Principles for evidence synthesis (15): (1) if evidence content is consistent, select the most straightforward language for expression; (2) if evidence content is complementary, merge based on logical relationships; (3) if evidence content is conflicting, prioritize evidence-based evidence, high-quality evidence, and the most recently published authoritative literature; (4) if evidence content is independent, retain the original description. The JBI Levels of Evidence and Grades of Recommendation (21) was used to grade the evidence based on the type of original literature from which it was derived.

## 3 Results

### 3.1 Search results

**Figure 1:**
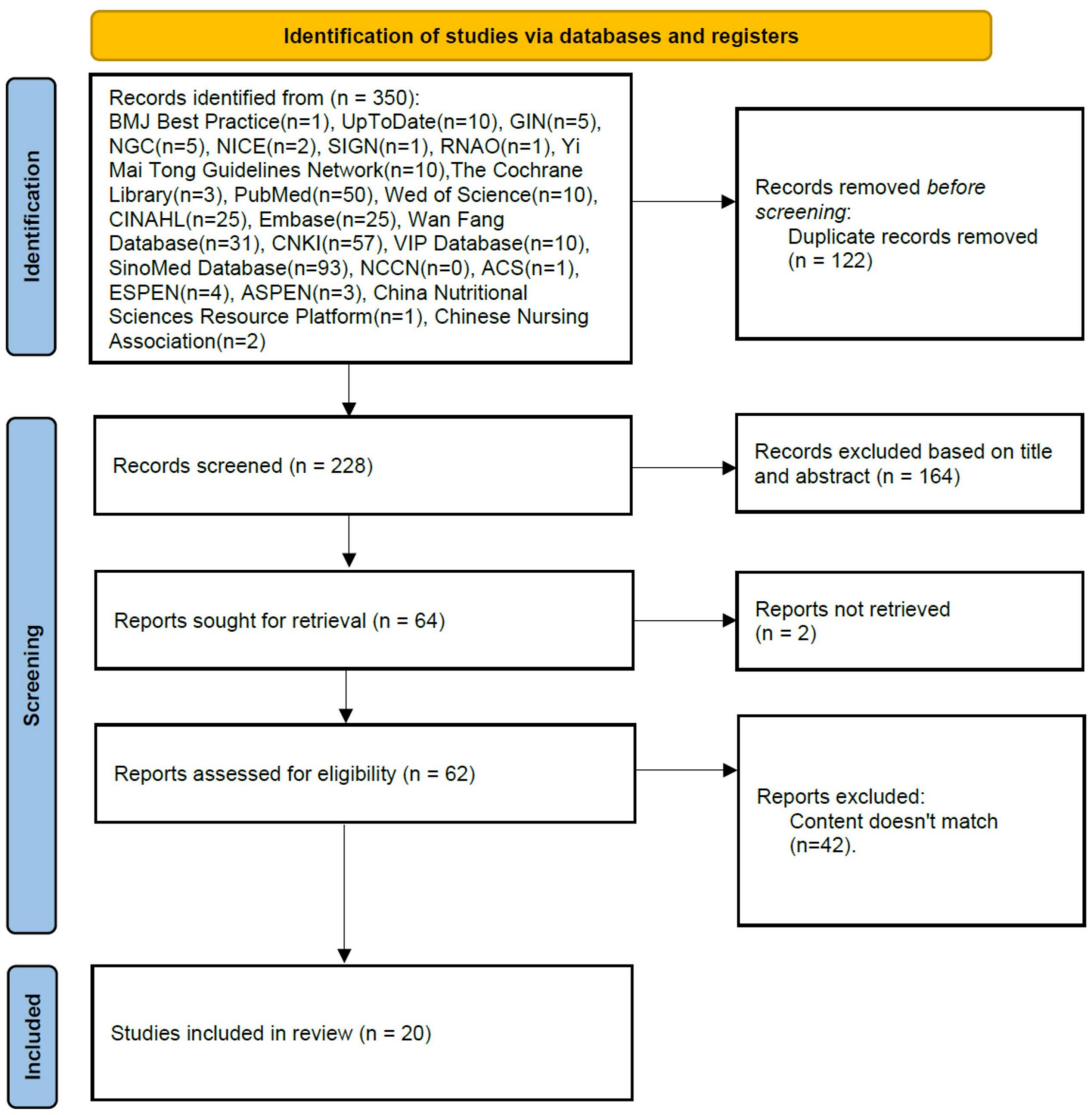
Literature screening flow chart. Creative Commons Attribution 4.0 International License (CC BY 4.0). Adapted from Page et al. (61)”, licensed under CC BY 4.0.

A total of 350 literature sources were retrieved in this evidence summary. Using EndNote software, 122 duplicate articles were removed, leaving a total of 228 articles. After reading the titles and abstracts, 164 articles that did not meet the requirements were excluded, leaving 64 articles. After reading the full text, 20 articles remained for further evaluation. These 20 articles include 2 clinical decisions (22, 23), 9 guidelines (24–32), 7 expert consensus statements (33–39), 1 evidence summary (14), and 1 systematic review (40). The document screening process is shown in Figure 1, and the general characteristics of the included articles are shown in Table 2.

**Table 2:** General characteristics of the included articles (*n* = 20).

Included literature	Literature reference	Year of publication	Type of literature	Literature theme
Hodin et al. (22)	UpToDate	2023	Clinical decision	Inpatient placement and management of nasogastric and nasoenteric tubes in adults
Romanowski et al. (23)	UpToDate	2024	Clinical decision	Nutritional demands and enteral formulas for adult surgical patients
Chinese Society for Parenteral and Enteral Nutrition (24)	Yi Mai Tong Guidelines Network	2017	Guideline	Nutritional support for tumor patients
Chinese National Cancer Center et al. (25)	Yi Mai Tong Guidelines Network	2023	Guideline	Perioperative diagnosis and treatment of esophageal cancer
Muscaritoli et al. (26)	ESPEN	2021	Guideline	Nutritional support for cancer patients
Weimann et al. (27)	ESPEN	2006	Guideline	Enteral nutritional support in surgical patients
Weimann et al. (28)	ESPEN	2021	Guideline	Nutritional support for surgical patients
NICE (29)	NICE	2017	Guideline	Adult tube-fed enteral nutrition support
Gkolfakis et al. (30)	PubMed	2021	Guideline	Management of perioperative tube-fed enteral nutrition in adult patients
Chinese Society of Nutritional Oncology et al. (31)	Wan Fang Database	2020	Guideline	Nutritional therapy for patients with esophageal cancer
Chinese Society for Parenteral and Enteral Nutrition (32)	CNKI	2023	Guideline	Parenteral enteral nutrition in adult patients
Chinese Nursing Association (33)	Chinese Nursing Association	2021	Expert consensus	Nursing care of adults on enteral nutritional support
Chinese Nursing Association (34)	Chinese Nursing Association	2021	Expert consensus	Retention and maintenance of nasoenteric tubes in adults
Chinese Society of Nutritional Oncology et al. (35)	Yi Mai Tong Guidelines Network	2023	Expert consensus	Management of enteral nutritional intolerance in oncology patients
Committee of Parenteral and Enteral Nutrition, Chinese Research Hospital Association (36)	Yi Mai Tong Guidelines Network	2024	Expert consensus	Clinical use of jejunostomy tubes
Chen et al. (37)	Web of Science	2018	Expert consensus	Nutritional therapy for patients with esophageal cancer
Oncology and Anesthesiology Group of Chinese Society of Anesthesiology et al. (38)	Wan Fang Database	2024	Expert consensus	Enhanced recovery strategies for patients undergoing surgery for esophageal cancer
Yu et al. (39)	Wan Fang Database	2024	Expert consensus	Perioperative enhanced recovery care for patients with esophageal cancer
Liu et al. (14)	CNKI	2023	Evidence summary	Perioperative nutritional management of patients with esophageal tumors
Li et al. (40)	Web of Science	2021	Systematic review	The effect of the use of jejunostomy versus nasoenteric tube for enteral nutrition after esophagectomy

### 3.2 Literature quality evaluation results

#### 3.2.1 Quality evaluation results of guidelines

A total of 9 guidelines were included (24–32). The standardized percentage scores across domains and their corresponding quality ratings are presented in Table 3.

**Table 3:** Clinical Yupal guidelines for quality evaluation results (*n* = 9).

Guidelines	Standardized scores in various domains (%)						≥60%	≥30%	Quality grade
	Scope and purpose	Stakeholder involvement	Rigor	Clarity	Applicability	Independence			
Chinese Society for Parenteral and Enteral Nutrition (24)	75.00	75.00	82.29	84.72	56.25	66.67	5	6	B
Chinese National Cancer Center et al. (25)	87.50	88.89	83.33	86.11	66.67	91.67	6	6	A
Muscaritoli et al. (26)	76.39	84.72	65.10	87.50	64.58	87.50	6	6	A
Weimann et al. (27)	58.33	50.00	60.93	86.11	64.58	37.50	3	6	B
Weimann et al. (28)	84.72	70.83	72.40	83.33	78.13	52.08	5	6	B
NICE (29)	98.61	79.17	68.75	84.72	67.71	39.58	5	6	B
Gkolfakis et al. (30)	88.89	79.17	76.04	88.89	59.38	100	5	6	B
Chinese Society of Nutritional Oncology et al. (31)	72.22	66.67	59.38	83.33	79.17	62.50	5	6	B
Chinese Society for Parenteral and Enteral Nutrition (32)	93.06	77.78	78.65	86.11	67.71	89.58	6	6	A

#### 3.2.2 Quality evaluation results of expert consensus

Seven expert consensus documents were included (33–36, 38, 39). For two of these documents (33, 34), item 1 (“Was the source of viewpoints clearly indicated?”) and item 5 (“Was reference made to other existing literature?”) were uniformly rated “No.” However, all other items received a “Yes” rating. The remaining five expert consensus documents received a “Yes” rating on all items. The overall quality of the seven consensus documents was assessed as satisfactory, and they were included.

#### 3.2.3 Quality evaluation results of systematic reviews

One systematic review (40) met all evaluation criteria (‘yes’ for all items), demonstrating good quality, and was therefore included.

#### 3.2.4 Quality evaluation results of clinical decisions and summary of evidence

Two clinical decision-making articles (22, 23) and one evidence summary (14) were included. These traced back to the following sources: one case–control study (41), one systematic review (42), and one expert consensus (43). For the case–control study by Yanjun et al. (41), the evaluation result for Item 7 (“Were measures taken to control for confounding factors?”) was ‘unclear’. For the systematic review by Patkova et al. (42), evaluation results were ‘unclear’ for Item 6 (“Was the quality assessment performed independently by two or more reviewers?”) and a response of ‘no’ was provided for Item 9 (“Was the possibility of publication bias assessed?”). All other items received a ‘yes’ rating. For the expert consensus by Rousseau et al. (43), the evaluation result was ‘yes’ for all items.

### 3.3 Summary and description of evidence

Following data extraction from the 20 included articles, all evidence statements related to perioperative tube-fed EN for patients with esophageal cancer were collated and organized. Two researchers independently performed thematic analysis of the extracted evidence. The initial themes were generated by grouping evidence statements with similar content. These were then refined through iterative discussion between the two researchers. Disagreements were resolved through consultation with a third researcher.

This process identified nine overarching themes that comprehensively cover the key aspects of perioperative EN management. These themes are organized according to the clinical pathway of nutritional support, from team establishment through to outcome evaluation. The nine themes include: team management; nutritional risk screening and assessment; indications for nutritional support; routes and timing of EN support; target nutrient requirements; EN formulas; implementation of tube feeding; tube care and maintenance; and monitoring and evaluating nutritional support outcomes. A total of 40 best evidence statements were summarized, as presented in Table 4.

**Table 4:** Summary of best evidence for nutritional support in esophageal cancer patients.

Evidence theme	Evidence content	Evidence level
Team management	1. Establish a multidisciplinary nutrition support team (including physicians, nurses, dietitians/nutritionists, clinical pharmacists, and rehabilitation therapists) to provide standardized nutritional care encompassing screening, assessment, diagnosis, intervention, and monitoring (29, 32).	5b
	2. A dedicated multidisciplinary nutrition support team should regularly monitor the therapeutic efficacy and complications of enteral nutrition support (30, 36).	5b
	3. All healthcare professionals directly involved in patient care should receive education and training relevant to their roles on the importance of providing adequate nutrition. This should include: ① Nutritional requirements and indications for nutritional support; ② Options for nutritional support (oral, enteral, parenteral); ③ Ethical and legal concepts; ④ Potential risks and benefits; ⑤ When and where to seek expert advice (29).	5c
Nutritional risk screening and assessment	4. Healthcare professionals should perform malnutrition risk screening with appropriate training (29).	5c
	5. Esophageal cancer is the malignancy associated with the highest risk of malnutrition. It is recommended to screen all diagnosed patients for nutritional risk using the NRS 2002 scale (31).	5b
	6. For esophageal cancer patients identified as at risk during screening, further nutritional assessment using the PG-SGA scale is recommended (31).	5b
	7. For patients identified as at risk during screening, an objective and quantitative assessment is recommended, including nutritional intake, stress level, inflammatory response, energy expenditure level, metabolic status, organ function, body composition analysis (including muscle mass and strength), physical fitness testing, and psychological status (26, 31, 32).	5b
	8. All hospitalized patients should undergo nutritional risk screening upon admission, and screening should be repeated weekly (26, 27, 29).	5b
Indications for nutritional support	9. Preoperative nutritional therapy for 7–14 days should be initiated if the patient has at least one of the following: NRS 2002 score ≥ 5, weight loss ≥ 10% within 6 months, BMI < 18.5 kg/m^2^, PG-SGA rating C, or serum albumin < 30 g/L (in the absence of hepatic or renal dysfunction) (31, 37, 38).	2c
	10. Postoperative nutritional therapy is recommended for all patients who benefit from preoperative nutritional therapy, malnourished patients, patients unable to eat orally postoperatively, or patients with oral intake meeting <60% of energy requirements 1 week postoperatively (24, 31).	5b
Routes and timing of enteral nutrition support	11. For patients undergoing esophageal cancer surgery, enteral nutrition is the preferred route (39). Based on medical experience and patient conditions, oral intake, nasogastric tube feeding, or jejunostomy tube feeding is recommended as an early postoperative route for enteral nutrition (25, 37).	1b
	12. Jejunostomy may be the preferred enteral nutrition route following esophagectomy (40).	1b
	13. Enteral nutrition should be initiated as early as possible after surgery (27), and can be administered within 24–48 h postoperatively (25, 30).	1a
	14. Tube feeding should be discontinued once the patient can consume sufficient food orally (29).	5b
Target nutrient requirements	15. For cancer patients, the target energy requirement is recommended to be based on the actual resting energy expenditure measured by indirect calorimetry. If measurement is unavailable, 25–30 kcal/kg/day (1 kcal = 4.18 kJ) is suggested (24, 32).	5b
	16. Initial nutritional support should not exceed 50% of the estimated target energy and protein requirements. Within 24–48 h postoperatively, nutritional support should be gradually increased based on metabolic status and gastrointestinal tolerance to meet full requirements. Full requirements for fluids, electrolytes, vitamins, and minerals should be provided from the start of feeding (29).	5b
Enteral nutrition formulas	17. Recommended macronutrient provision: ① Protein: 1.2–2 g/(kg·d); ② Glucose: 1–2 g/(kg·d), providing approximately 50% of energy; ③ Fat: 1–1.2 g/(kg·d), providing ≤15% of energy (42).	5a
	18. Recommended micronutrient provision: Supplement vitamins A, C, and D with a standard multivitamin preparation (43). Vitamin and mineral supplementation should approximate the recommended daily intake; high-dose micronutrient supplementation is not encouraged without specific indications (24, 26).	1b
	19. Immuno-modulating substrates (arginine, omega-3 fatty acids, and nucleotides) are recommended, regardless of nutritional risk. Whenever possible, these formulations should be initiated 5–7 days before surgery and continued postoperatively for 5–7 days after uncomplicated surgery (27, 32).	1b
	20. The osmolarity of the nutritional formula should be less than 330 mmol/L to reduce the incidence of diarrhea (35).	5b
	21. Increasing soluble fiber content in nutritional formulas can reduce the incidence of diarrhea (35). Based on slowing the feeding rate, reducing the concentration of the enteral nutrition formula, and adjusting the temperature to an appropriate level, it is recommended to add soluble fiber, with the option to include probiotics as well (35).	5b
	22. The use of probiotics may help protect gastrointestinal integrity and improve tolerance; however, current evidence remains insufficient to draw firm conclusions (35).	5b
	23. Standard whole-protein formulas are suitable for most patients and are recommended when initiating enteral nutrition (27, 32).	1a
Implementation of tube feeding	24. For postoperative patients, tube feeding should be administered via continuous infusion using an enteral feeding pump (35).	5b
	25. For stable patients with good tolerance receiving long-term tube feeding, intermittent infusion is recommended to restore a normal dietary rhythm. If intolerance occurs, pause or reduce the infusion rate to the previously tolerated level before gradually increasing it again, or switch from intermittent to continuous infusion (32).	5b
	26. Elevate the head of the bed to 30°–45° and maintain a semi-upright position for 30 min after feeding (35).	5b
	27. Enteral nutrition should be initiated at a low flow rate (10–20 mL/h). Due to limited intestinal tolerance, the rate should be increased cautiously; achieving the target rate may take 5–7 days (26).	1a
	28. Enteral nutrition should be started at low doses and concentrations, following a stepwise progression. A warmer should be used to maintain the formula temperature at 37–40 (24, 35).	5b
	29. Patient tolerance to enteral nutrition should be assessed every 4–6 h (32, 33), preferably using an enteral nutrition tolerance scoring tool.	3e
	30. Diarrhea occurring during tube feeding should first prompt the exclusion of disease-related or non-nutrition medication-related causes before considering discontinuation of enteral nutrition (32).	4b
Tube care and maintenance	31. The feeding tube, its securing device, tube patency, and the condition of the skin and mucosa at the fixation site should be checked daily (33).	5b
	32. The skin around the jejunostomy tube should be regularly disinfected, and dressings should be changed to keep the surrounding skin clean and dry (30, 33).	5b
	33. Nasoenteric tube function should be routinely checked by flushing every 4–8 h (22); jejunostomy tube function should be checked by flushing every 4–6 h (36). Gastric residual volume monitoring does not apply to nasoenteric or jejunostomy tubes and should be avoided to prevent tube clogging.	3d
	34. Flush the tube with 20–30 ml of warm water using a pulsed method before and after feeding, before and after medication administration, and if the tube has been clamped for more than 24 h (33, 34).	5b
	35. Solid medications should be thoroughly crushed and dissolved before administration (34); liquid medications are preferred (30).	5b
	36. Avoid mixing liquid medications with a pH ≤ 5 with the nutritional formula (33).	5b
	37. If the feeding tube is obstructed, flush it with 20–30 mL of warm water using aspiration and pulsed flushing. If ineffective, flush with 20–30 mL of a 5% sodium bicarbonate solution (33).	5b
	38. For obstructed nasoenteric tubes, connect a three-way connector to the tube. Attach an empty 10 mL syringe to one port and a 10 mL syringe filled with normal saline to the other port. Repeatedly aspirate outward by rotating the three-way valve. Use medication to clear the obstruction as prescribed. Never attempt to clear the tube by inserting a guidewire directly (34).	5b
Monitoring and outcome evaluation of nutritional support	39. Following esophageal cancer surgery, monitor body weight, complete blood count, electrolytes, liver and kidney function, albumin, and prealbumin 1–2 times per week, or daily if necessary (39).	5b
	40. During and after esophageal cancer treatment, clinicians/dietitians should regularly evaluate the efficacy of nutritional therapy to provide a basis for adjusting the nutritional treatment plan (31, 39).	5b

Evidence levels are based on the Joanna Briggs Institute Levels of Evidence and Grades of Recommendation (21).

According to the JBI Levels of Evidence, and based on the aggregation of sublevels (e.g., 1a–1b, 3a–3b, 5b–5c), the majority of evidence statements were classified as Level 5 (*n* = 29, 72.5%), primarily derived from expert consensus and guideline-based sources. The remaining evidence was grouped into higher-level evidence (Levels 1–2) and intermediate-level evidence (Levels 3–4) for the purpose of presentation, accounting for 20.0% (*n* = 8) and 7.5% (*n* = 3), respectively.

## 4 Discussion

### 4.1 Establishing a multidisciplinary team for professional perioperative nutrition management, accurate screening and assessment of nutritional risk, and preoperative nutritional therapy

Evidence items 1–3 summarize the establishment and training of the multidisciplinary nutrition support team (MDT). The nurse-led multidisciplinary team approach has been applied to perioperative care for cancer patients (44, 45). Key factors for the effectiveness of an MDT include stable relationships among team members, effective communication and leadership, and organizational support (46). In the perioperative nutrition management of esophageal cancer patients, an MDT can mobilize experts from multiple fields to collaborate, enabling continuous dynamic screening and assessment of nutritional status. This in turn develops personalized nutritional support plans, effectively improving patients’ nutritional status, promoting rapid postoperative recovery of gastrointestinal function, reducing postoperative complications, and lowering hospitalization costs (47).

Evidence items 4–8 summarize the tools and timing for nutritional risk screening and assessment. Cancer-specific symptoms and the management of malnutrition in esophageal cancer patients are current areas of focus. Patients often present with symptoms such as dysphagia, indigestion, nausea, and vomiting, leading to reduced food intake (48). Preoperative malnutrition and sarcopenia are risk factors for postoperative complications (49). Following surgery, patients’ quality of life and nutritional status can be significantly impacted by esophageal cancer treatment (50). Therefore, perioperative nutritional risk screening and assessment are crucial for in-hospital treatment and even home-based rehabilitation after discharge.

Evidence items 9–10 summarize the indications for preoperative and postoperative nutritional support. Based on the results of nutritional screening and assessment, dietary advice and nutritional support should be provided preoperatively. If oral intake fails to meet target requirements, tube feeding (EN), supplemental parenteral nutrition, or total parenteral nutrition may be initiated. Research (51) indicates that extending preoperative nutritional support is safe and feasible, potentially improving physical function, alleviating fatigue, and enhancing quality of life in patients after esophagectomy. Multiple expert consensuses (37, 38) recommend initiating nutritional therapy if the preoperative NRS 2002 score is ≥5. Guidelines (31) suggest that preoperative nutritional therapy for 7 to 14 days should be provided if at least one of the following criteria is met: weight loss ≥10% within 6 months, BMI < 18.5 kg/m^2^, PG-SGA rating C, or serum albumin <30 g/L (in the absence of hepatic or renal dysfunction).

### 4.2 Initiating tube feeding early postoperatively and setting individualized nutrient requirements

Evidence items 11–14 summarize the routes and timing for initiating EN support via tube feeding. Early postoperative initiation of nutritional support is vital to maintain the state achieved during prehabilitation and to prevent malnutrition-related complications (52). Early tube feeding in postoperative esophageal cancer patients can improve nutritional status, promote wound healing, reduce complications, and shorten hospital stay (31). Articles (53) demonstrate that initiating EN within 24–48 h postoperatively does not significantly impact 30-day mortality, feeding intolerance, gastrointestinal complications, or pneumonia rates. Therefore, tube feeding can be safely initiated within this timeframe. Jejunostomy tubes and nasoenteric tubes are two common routes. Research indicates that jejunostomy tubes are associated with lower rates of postoperative pneumonia, shorter hospital stays, fewer feeding interruptions, and fewer tube dislodgments than nasoenteric tubes (40). Consequently, jejunostomy may be the more suitable feeding route post-esophagectomy, and EN can commence within 24 h of tube placement (30).

Evidence items 15–16 summarize daily target nutrient requirements. Guidelines (24, 32) recommend that the target energy intake for esophageal cancer patients be based on resting energy expenditure measured by indirect calorimetry. However, as this equipment is not universally available, an intake of 25–30 kcal/kg/day (1 kcal = 4.18 kJ) is suggested. Within the first 24–48 h postoperatively, intake should be gradually increased to the target level based on gastrointestinal tolerance, starting at no more than 50% of the estimated target energy and protein requirements (29). Nurses can calculate the daily energy intake based on each patient’s weight and closely monitor tolerance during the initial phase of tube feeding, gradually increasing the infusion rate to meet the target needs.

Evidence items 17–23 summarize aspects related to EN formulas. Clinical practice guidelines (23) recommend that EN should provide protein at 1.2–2 g/(kg·d), glucose at 1–2 g/(kg·d), and fat at 1–1.2 g/(kg·d). Other guidelines (31) suggest that a higher protein intake target of 1.5–2.0 g/(kg·d) may yield more optimal results. In postoperative esophageal cancer patients, formulas supplemented with immunomodulatory substrates (arginine, n-3 fatty acids, nucleotides) can significantly reduce the incidence of incisional bleeding, wound infection, and pulmonary infection while improving nutritional status (54). This concept aligns with the emerging understanding that nutrition, immunity, inflammation, and prognosis form an interconnected loop, as recently demonstrated in head and neck cancer research (55). Thus, the selection of specific nutrients should be tailored to the patient’s clinical condition.

### 4.3 Standardizing EN administration and optimizing tube care procedures

Evidence items 24–30 summarize the methods for administering tube feeding. The Nursing Group Standard for Adult EN Support (33) describes three infusion methods: bolus administration, intermittent gravity drip, and continuous pump infusion. Compared to bolus or gravity drip, continuous pump infusion reduces the incidence of diarrhea, improves glycemic control, and enhances nutritional status by increasing prealbumin levels (56).

Diarrhea is a common complication of postoperative EN in esophageal cancer patients, with reported incidence rates as high as 31.69% in some Chinese hospital settings (57). Research (58) has identified risk factors for EN-associated diarrhea (ENAD), including EN duration, elevated blood urea nitrogen levels, daily formula volume, probiotic use, and an admission diagnosis of respiratory disease. The Hart Diarrhea Score (59) can assist nurses in identifying ENAD based on stool consistency and volume. Guidelines (24, 26) and expert consensus (35) recommend starting with low doses, concentrations, and infusion rates, gradually increasing based on intestinal tolerance, and using a warmer to maintain formula temperature at 37–40 °C. Moreover, formulas with increased soluble fiber, probiotics, and an osmolarity below 330 mmol/L (35) are suggested to prevent ENAD.

Evidence items 31–38 summarize tube care and maintenance procedures. Tube occlusion is a significant adverse event. To prevent this, nasoenteric tubes should be flushed every 4–8 h (22), and jejunostomy tubes every 4–6 h (36), allowing nurses to standardize procedures. Additionally, flush the tube with 20–30 mL of warm water (25–35 °C) using a pulsed method before and after feeding, before and after medication administration, and whenever the tube is clamped for more than 24 h (33, 34, 60) to ensure patency, prevent contamination, and reduce infection risk. If occlusion occurs, flushing with 20–30 mL of 5% sodium bicarbonate solution is recommended after unsuccessful attempts with warm water (33). If both methods fail, research (60) suggests instilling a solution of pancreatic enzymes dissolved in water with 8.4% sodium bicarbonate, clamping for 5–10 min, then flushing.

Evidence items 39–40 summarize the monitoring of parameters and the evaluation of efficacy during tube-feeding support. During perioperative nutritional therapy for patients with esophageal cancer, efficacy should be assessed regularly. Furthermore, the treatment plan was adjusted promptly through communication with physicians (31, 39).

## 5 Limitations

First, although a comprehensive search strategy was employed, some relevant studies may have been missed, particularly those published in languages other than English or Chinese or in non-indexed journals. Second, the quality of the included evidence varied. According to the JBI Levels of Evidence used in this study, a substantial proportion of the evidence statements were classified as Level 5 (72.5%), mainly derived from expert consensus and guideline-based sources, whereas only a limited number of statements were supported by evidence from more rigorous study designs. This distribution reflects the current limitations of the evidence base in this field. Therefore, recommendations based on lower-level evidence should be interpreted with caution in clinical practice. Third, this evidence summary focused specifically on perioperative tube-fed EN in patients with esophageal cancer; the findings may not be generalizable to other patient populations or to other types of nutritional support.

## 6 Conclusion

This evidence summary summarizes 40 evidence-based recommendations across nine domains. These include team management, nutritional risk screening and assessment, indications for nutritional support, routes and timing of EN support, target nutrient requirements, EN formulas, implementation of tube feeding, tube care and maintenance, and monitoring and outcome evaluation of nutritional support. Thus, this provides evidence-based guidance for healthcare professionals implementing perioperative tube feeding in patients with esophageal cancer. Notably, clinicians can select and apply these recommendations based on local healthcare resources and individual patient conditions to deliver scientifically grounded and efficient nutritional support.

## Data Availability

The original contributions presented in the study are included in the article/Supplementary material, further inquiries can be directed to the corresponding author.
