# Supplementary material for: Systematic review of the management of perioperative tube-fed enteral nutrition in esophageal cancer patients
Source: Front Nutr. 2026 Jul 24;13:1717313. doi: 10.3389/fnut.2026.1717313 (PMC13447076; doi:10.3389/fnut.2026.1717313)
Supplement: Supplementary file 1 [file Table_1.docx]

| **Database/Website** | **Search Terms** |
| --- | --- |
| PubMed | #1: (((((((((((((((esophageal neoplasms[MeSH Major Topic]) OR (esophageal neoplasms[Title/Abstract])) OR (Esophageal Neoplasm[Title/Abstract])) OR (Esophagus Neoplasm[Title/Abstract])) OR (Esophagus Neoplasms[Title/Abstract])) OR (Cancer of Esophagus[Title/Abstract])) OR (Esophageal Cancer[Title/Abstract])) OR (Esophageal Cancers[Title/Abstract])) OR (Cancer of the Esophagus[Title/Abstract])) OR (Esophagus Cancer[Title/Abstract])) OR (Esophagus Cancers[Title/Abstract])) OR (esophagectomy[MeSH Major Topic])) OR (esophagectomy[Title/Abstract])) OR (Esophagectomies[Title/Abstract])) OR (esophageal surgery[Title/Abstract])) OR (esophageal resection[Title/Abstract])  #2: (((((((((((((enteral nutrition[MeSH Major Topic]) OR (enteral nutrition[Title/Abstract])) OR (Tube Feeding[Title/Abstract])) OR (Enteral Feeding[Title/Abstract])) OR (Gastric Feeding Tubes[Title/Abstract])) OR (Gastric Feeding Tube[Title/Abstract])) OR (Force Feeding[Title/Abstract])) OR (Force Feedings[Title/Abstract])) OR (nutrition therapy[MeSH Major Topic])) OR (nutrition therapy[Title/Abstract])) OR (Medical Nutrition Therapy[Title/Abstract])) OR (Nutritional Support[MeSH Major Topic])) OR (Nutritional Support[Title/Abstract])) OR (Artificial Feeding[Title/Abstract])  #3: (((((guideline[Title/Abstract]) OR (consensus[Title/Abstract])) OR (meta-analysis[Title/Abstract])) OR (system review[Title/Abstract])) OR (evidence summary[Title/Abstract])) OR (best practice[Title/Abstract])  #4: #1 AND #2 AND #3 |
| Embase | ('esophageal neoplasms':ab,ti OR 'esophagus neoplasm':ab,ti OR 'cancer of esophagus':ab,ti OR 'esophagus cancer':ab,ti OR 'esophageal cancers':ab,ti OR 'esophagectomy':ab,ti OR 'esophagectomies":ab,ti OR 'esophageal surgery':ab,ti OR 'esophageal resection':ab,ti) AND ('enteral nutrition':ab,ti OR 'enteral feeding':ab,ti OR 'nutrition therapy':ab,ti OR 'nutritional support':ab.ti OR 'artificial feeding':ab,ti) AND ('guideline':ab,ti OR 'consensus':ab,ti OR 'meta-analysis':ab,ti OR 'system review':ab,ti OR 'evidence summary':ab,ti OR 'best practice':ab.ti) |
| Cochrane Library | esophageal neoplasms OR esophagus neoplasm OR cancer of esophagus OR esophagus cancer OR esophageal cancers OR esophagectomy OR esophagectomies OR esophageal surgery OR esophageal resection in Title Abstract Keyword AND enteral nutrition OR enteral feeding OR nutrition therapy OR nutritional support OR artificial feeding in Title Abstract Keyword - (Word variations have been searched) |
| Web of Science | #1: TI=(esophageal neoplasm* OR Esophagus Neoplasm* OR Cancer of Esophagus OR Esophageal Cancer* OR Cancer of the Esophagus OR Esophagus Cancer* OR esophagectomy OR Esophagectomies OR esophageal surgery OR esophageal resection)  #2: TI=(guideline OR consensus OR meta-analysis OR system review OR evidence summary OR best practice)  #3: TI=(enteral nutrition OR Tube Feeding OR Enteral Feeding OR Gastric Feeding Tube* OR Force Feeding* OR nutrition therapy OR Medical Nutrition Therapy OR Nutritional Support OR Artificial Feeding)  #4: #3 AND #2 AND #1 |
| CINAHL | S1: esophageal neoplasms OR esophagus neoplasm OR cancer of esophagus OR esophagus cancer OR esophageal cancers OR esophagectomy OR esophagectomies OR esophageal surgery OR esophageal resection  S2: enteral nutrition OR enteral feeding OR nutrition therapy OR nutritional support OR artificial feeding  S3: guideline OR consensus OR meta-analysis OR system review OR evidence summary OR best practice  S4: S1 AND S2 AND S3 |
| CNKI/ WanFang | 主题=(食管癌 OR 食道癌 OR 食管肿物 OR 食管切除术 OR 食管癌根治术 OR 食管手术) AND 主题=(营养管理 OR 营养支持 OR 肠内营养 OR 人工喂养) AND 主题=(指南 OR 共识 OR 标准 OR 证据总结 OR 最佳实践 OR 系统评价 OR Meta) |
| VIP | 题名或关键词=(食管癌 OR 食道癌 OR 食管肿物 OR 食管切除术 OR 食管癌根治术 OR 食管手术) AND题名或关键词=(营养管理 OR 营养支持 OR 肠内营养 OR 人工喂养) AND 题名或关键词=(指南 OR 共识 OR 标准 OR 证据总结 OR 最佳实践 OR 系统评价 OR Meta) |
| SinoMed | ("食管癌"[常用字段:智能] OR "食道癌"[常用字段:智能] OR "食管肿物"[常用字段:智能] OR "食管切除术 OR食管癌根治术 OR 食管手术"[常用字段:智能]) AND("营养管理"[常用字段:智能] OR "营养支持"[常用字段:智能] OR "肠内营养"[常用字段:智能] OR "人工喂养"[常用字段:智能]) AND("指南"[常用字段:智能] OR "共识"[常用字段:智能] OR "标准"[常用字段:智能] OR "证据总结"[常用字段:智能] OR "最佳实践"[常用字段:智能] OR "系统评价"[常用字段:智能] OR "Meta"[常用字段:智能]) |
| NCCN / ACS / ESPEN / ASPEN / Others | Manual search for guidelines and consensus documents |

**Note:** Search strategies for Chinese databases (CNKI, WanFang, VIP, and SinoMed) were conducted using equivalent Chinese subject terms and free-text keywords corresponding to the English search strategy. These search strings are presented in their original language to ensure accuracy and reproducibility.
